# Crustacean hyperglycemic hormone is synthesized in the eyestalk and brain of the crayfish *Procambarus clarkii*

**DOI:** 10.1371/journal.pone.0175046

**Published:** 2017-04-03

**Authors:** Rosaura Loredo-Ranjel, María Luisa Fanjul-Moles, Elsa G. Escamilla-Chimal

**Affiliations:** Laboratorio de Neurofisiología y Ritmos Biológicos, Departamento de Ecología y Recursos Naturales, Facultad de Ciencias, Universidad Nacional Autónoma de México, Mexico City, Mexico; International Centre for Genetic Engineering and Biotechnology, ITALY

## Abstract

Crustacean hyperglycemic hormone (CHH) is a neuropeptide that is synthesized, stored, and released by brain and eyestalk structures in decapods. CHH participates in the regulation of several mechanisms, including increasing the level of glucose in hemolymph. Although CHH mRNA levels have been quantified and the CHH protein has been localized in various structures of the crayfish *P*. *clarkii*, CHH synthesis has only been reported in the X-organ-sinus gland (XO-SG). Therefore, the aim of this study was to use *in situ* hybridization to determine whether CHH mRNA is located in other structures, including the putative pacemaker, eyestalk and brain, of crayfish *P*. *clarkii* at two times of day. CHH mRNA was observed in both the eyestalk and the brain of *P*. *clarkii*, indicating that CHH is synthesized in several structures in common with other crustaceans, possibly to provide metabolic support for these regions by increasing glucose levels.

## Introduction

The metabolic neuropeptide crustacean hyperglycemic hormone (CHH) is pleiotropic and is mainly synthesized, stored and released by neuroendocrine cells in the medulla terminalis (MT) of the X-organ (XO) in the crustacean eyestalk. In decapod crustaceans, the axons of the XO project distally along the ventrolateral margin of the eyestalk until they form a neurohemal organ called the sinus gland (SG). Serotonergic inputs to the MTXO are thought to invoke the release of CHH into the circulation via the ophthalmic artery [1], [2]. This hormone is mainly involved in regulating glucose levels in the hemolymph, although the participation of CHH in other physiological processes, such as molting, reproduction and osmoregulatory responses to stress, is well established (for review, see [3], [4]).

Although the XO–SG complex is considered the main locus of neuropeptide production, CHHs have also been observed in several neural tissues, including the pericardial organs [5], [6], [7], cerebral ganglia [8], ventral nerve cord [9], and retinal tapetal cells [10], and non-neural tissues such as the foregut and hindgut [11]. CHH activity varies during the life cycle of decapod crustaceans and contributes to mechanisms underlying adaptations to stressful conditions through a dual-feedback control system [12]. Clear circadian changes in CHH secretion and release [13], [14] have been associated with circadian changes in glucose levels in the hemolymph [15] in the crayfish *Astacus leptodactylus*. In other decapods, such as *Procambarus clarkii*, the circadian modulation of CHH has been described in the crayfish eyestalk and retina [16]. Recently, these authors investigated the expression dynamics of CHH mRNA in the crayfish *Procambarus clarkii* over a day/night cycle under both entrained LD and free-running (DD) conditions [8]. Their data provided evidence of a circadian rhythm of CHH transcription. Intriguingly, using immunocytochemistry, they also found that CHH was expressed in the central brain in several proto- and tritocerebral cell clusters known to express canonical clock proteins, such as Timeless, Period and Clock (Bmal) [8], [17]. These observations, in combination with the finding that CHH immunoreactivity was reported in both the cytoplasm and nuclei of brain cells (similar to expression during circadian clock transcriptional repressor nuclear translocation events), suggested that there is a possible relationship between the central circadian clock and CHH.

It has been proposed that the circadian rhythms of *Procambarus clarkii* are controlled by a multi-oscillatory distributed system that includes three pairs of coupled oscillators (i.e., the retina, the XO-SG complex of the eyestalk, and the central brain). However, we do not fully understand how the different pacemakers of this system connect, synchronize and undergo entrainment [18]. We have yet to identify the signaling molecules and neural mechanisms that govern the ability of the pacemakers to maintain the biological timing system. Based on the results of previous studies, we propose that CHH is a possible mediator in this system. Although it has been demonstrated that CHH is synthesized in the retina [1] and XO [19], [20], neither its synthesis nor the presence of its transcripts have been observed in the various brain cells that express clock proteins. Thus, in this study, we localized the position of CHH-positive cells at two points during the circadian rhythm that were previously described as crucial for CHH transcriptional rhythm [8]. We demonstrate the presence of CHH mRNA in both the various cell clusters of the eyestalk and the brain of crayfish *P*. *clarkii*, where clock protein expression was previously described. Both the cytoplasm and the nuclei of these cells expressed differential CHH positivity that was associated with the time of day. Our results suggest that CHH is synthesized in the different pacemakers of *P*. *clarkii*, indicating that in this decapod, CHH transcription is likely to be controlled by the circadian clock. These data contribute to the body of knowledge related to the complex circadian system of crustaceans.

## Materials and methods

### Ethical statement

All experiments were performed in accordance with the current laws of Mexico, the country in which they were conducted. This work did not involve endangered or protected species. On the contrary, this crustacean is considered an invasive species, and no specific permission was therefore required for the use of these locations/activities. This protocol was approved by the Research Ethics Committee of the Faculty of Medicine-UNAM and assigned the number CONBIOÉTICA 09CEI066201403212. All animals were maintained under optimal laboratory conditions to ensure animal welfare. The crayfish were anesthetized by hypothermia to avoid animal suffering and then sacrificed via decapitation before the dissections were performed.

### Animals

Adult *Procambarus clarkii* crayfish were collected from streams near the Conchos River, Chihuahua, Mexico. They were kept in aquaria, acclimatized to laboratory conditions for 15 days at 20 ± 1°C, pH 7.9, and 5.7 mg/l O_2_, fed *ad libitum* with Camaronina (35%; Purina Pro Plan, Sociétédes Produits Nestlé S.A.; Vevey, Switzerland), and exposed to a 12:12 light–dark cycle (LD). Neon lamps that provided low-intensity light (0.043 W/m^2^) were turned on at 7:00 h (Zeitgeber Time, ZT0) and off at 19:00 h (ZT12). The aquaria contained polyvinyl tubes that were used to simulate burrows, thereby allowing the animals to hide from the light. The experimental protocols adhered to the guidelines of the Declaration of Helsinki and other international ethical guidelines [21].

### RNA extraction

Twelve crayfish were decapitated at ZT1, and their heads were then dissected. The brain and eyestalk were removed, placed in cold RNAlater (Ambion, Life Technologies Corp.; Carlsbad, CA, USA), and immediately transferred to an Eppendorf tube containing 500 μl of QIAzol (Qiagen Sciences; Maryland, USA). The samples were homogenized using a syringe and then vortexed. Total RNA was extracted using cold TriPure (F. Hoffmann-La Roche AG; Basel, Switzerland) according to the manufacturer's instructions and following the methodology recommended in the protocol published by Chomczynski and Sacchi [22]. Chloroform (Amresco Inc.; Solon, Ohio, USA) was added to separate DNA and RNA from proteins. The aqueous phase was transferred to another Eppendorf tube, and RNA was precipitated by adding 100% isopropanol (Amresco Inc.; Solon, Ohio, USA). After the samples were centrifuged, the pellets were washed in 75% ethanol at -20°C. The samples were centrifuged again, decanted in ethanol and dried into pellets. Finally, the samples were hydrated and stored at -20°C.

### Reverse Transcription (RT-PCR)

To obtain cDNA, reverse transcription was performed using 0.5 μg of Oligo (dT) 15 Primer (Promega Corporation; Madison, WI, USA), 2 μg of total RNA and 2 μl of sterile water. The mixture was heated to 70°C for 5 min. It was then placed on ice, and the following reagents were added: 5 μl of 5x buffer M-MLV-RT (Promega Corporation), dNTP mix, each nucleotide at a final concentration of 0.5 mM (Altaenzymes; Alberta, Canada), 40 units of RNasin recombinant ribonuclease inhibitor (Promega Corporation), 8 μl of sterile water and 200 units of reverse transcriptase M-MLV (Promega Corporation). The samples were incubated for one hour at 42°C in a Techne TC-312 thermocycler (Techne, Bibby Scientific Ltd.; Staffordshire, UK). The reverse transcriptase was inactivated by incubating the mixture at 70°C for 15 min.

### PCR (Polymerase Chain Reaction)

The following reagents were used to amplify PCR products: 1 μl of 10x Taq reaction buffer (Altaenzymes), dNTP mix with each nucleotide at a final concentration of 2 mM (Altaenzymes), 2.3 mM MgCl_2_ (Altaenzymes), each primer at 500 nM [8], 2 μl of cDNA that was synthesized from the brain and eyestalk, 3.5 μl of sterile water and 0.1 units of Taq DNA polymerase (Altaenzymes). After the samples were centrifuged, two drops of mineral oil were added to each tube to prevent the evaporation of the mixture. The samples were incubated in a Techne thermal cycler (Techne, Bibby Scientific Ltd.; Staffordshire, UK) under the following conditions: initial denaturation at 94°C for 2 min followed by 40 cycles of denaturation for 1 min at 94°C, elongation for 45 s at 60°C and extension for 1 min at 72°C and a final extension for 10 min at 72°C.

The fragment length was verified on 1.2% agarose gels and purified using a QIAEX II Gel Extraction Kit 150 (Qiagen GmbH; Hilden, Germany) according to the manufacturer’s instructions. The fragment was sequenced at the Unidad de Proteogenómica, Instituto de Neurobiología, Universidad Nacional Autónoma de México (Juriquilla, Querétaro, Mexico).

### Synthesis of the probe

The synthesis and labeling of the probe was carried out using DIG-NICK translation mix (Roche Diagnostics GmbH, Werk Penzberg; Penzberg, Germany) and 1 μg of sample (previously purified using the Qiagen kit), 4 μl of DIG-NICK (Roche) and water to reach a final volume of 20 μl. The mixture was incubated at 15°C for 180 min. The reaction was stopped using 5 μl of EDTA (0.5 M, pH 8) and then heated at 65°C for 10 min. Next, 5 μl of 3 M sodium acetate and 25 μl of absolute ethanol (-20°C) were added, and the mixture was incubated for 15 min at -70°C. The mixture was centrifuged at 10,900 rpm for 10 min and then decanted. A further 5 μl of 3 M sodium acetate and 25 μl of absolute ethanol (-20°C) were then added. The mixture was then centrifuged for 10 min, decanted, hydrated with sterile water and stored at -20°C after it was verified using 1.2% agarose gel electrophoresis.

### Tissue processing

Ten crayfish were used in this experiment. The dissections were performed at ZT1 and ZT16, which were times chosen according to the data described in Tensen et al. [23] and Nelson-Mora et al. [8]. Samples of the brain, optic lobe and retina were fixed in 4% paraformaldehyde for 24 h at 4°C. They were then washed with PBS and cryopreserved in 20% and then 30% sucrose. Sections were cut on a cryostat (Leica Biosystems CM 1510S; Nussloch, Germany) at a thickness of 10 μm.

### *In situ* hybridization

The slices were washed with phosphate-buffered saline (PBS) and incubated for 48 hours at 4°C with 60 ml of PBS, 1.2 g of bovine serum albumin (Sigma-Aldrich, St. Louis, MO, USA), 3 g of milk powder and 1200 μl of Triton X-100 (Golden Bell reagents, Mexico, DF). Then, they were washed with deionized water and labeled using a Super Pap pen (Biocare Medical, Inc., Concord, CA, USA). To detect expression using *in situ* hybridization, an alkaline phosphatase kit (RISH AP) was used (Chromogenic Detection of Labeled Probes Digoxigenin, Biocare Medical, Inc., Concord, CA, USA), with some modifications to the protocol. Then, the samples were washed, and protein was digested by adding 200 μl of digestion reagent (1:2) to each slice. The slices were then incubated for 1 min at room temperature and washed with deionized water. Recovery was performed using 10x RISH retrieval buffer (Biocare Medical, Inc.). For *in situ* hybridization, 20 μl of the probe, 20 μl of 5% bovine serum albumin (Sigma-Aldrich) and 40 μl of deionized formamide (Amresco, Inc., Solon, OH, USA) were combined, and 50 μl of this mixture was added to each slide. For the control, 20 μl of sterile water, 20 μl of 5% bovine serum albumin and 40 μl of deionized formamide (Amresco, Inc.) were combined, and 50 μl of this mixture was added to each slide. The slides were incubated for 1 hour at 60°C in a humid chamber. Subsequently, the samples underwent several washes in Tris-buffered saline (TBS), and a RISH secondary reagent (Biocare Medical, Inc.) was then added. The slides were then incubated for 15 min. The samples were washed with TBS, and 4 drops of RISH AP secondary reagent (Biocare Medical, Inc.) was then added. The slides were then incubated for 15 min. The samples were washed again with TBS, 4 drops of prepared red Warp containing 17.5 μl of chromogen and 1250 μl of buffer were added (Biocare Medical, Inc.), and the slides were incubated for 10 min. The samples were then washed in deionized water, stained with Toluidine blue, washed again with deionized water, allowed to dry, and mounted using Eco Mount (Biocare Medical, Inc.).

### Image processing

Images were obtained using a microscope (Olympus Provis AX70; Tokyo Japan) with Q-capture Pro7 software and cropped, resized and adjusted for brightness and contrast using CorelDRAW software.

## Results and discussion

The probe for CHH mRNA was obtained using samples that were collected from the eyestalk and brain at ZT1. Using *in situ* hybridization, a positive signal for CHH mRNA was observed in both the eyestalk and the brain of the crayfish *P*. *clarkii*.

**Fig 1:**
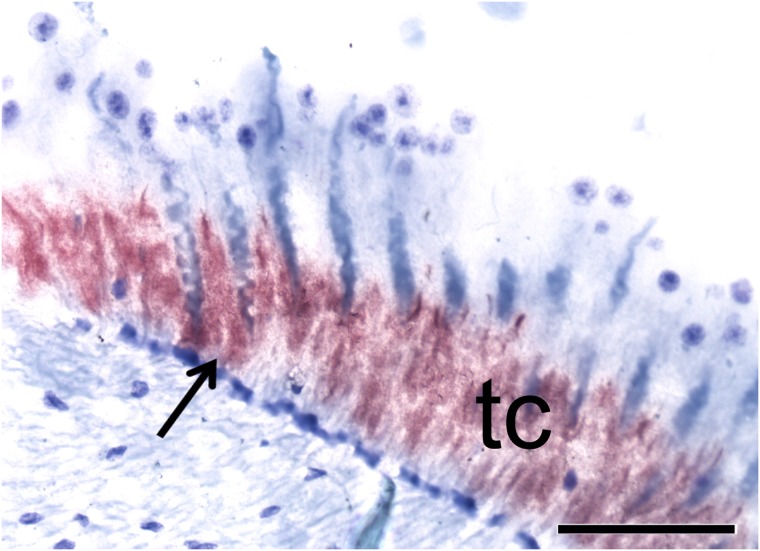
Retina of crayfish *P*. *clarkii* at ZT1. Photomicrographs showing CHH mRNA signal (arrows) in tapetal cells. Scale bar, 100 μm.

As shown in Fig 1, the retina showed a positive signal for CHH mRNA in tapetal cells, as expected based on previous studies produced by our laboratory, in which the CHH protein was found to be localized in this area at ZT1. The finding that CHH is synthesized in tapetal cells is important because these cells are responsible for providing metabolic support and increasing glucose levels in the retina.

**Fig 2:**
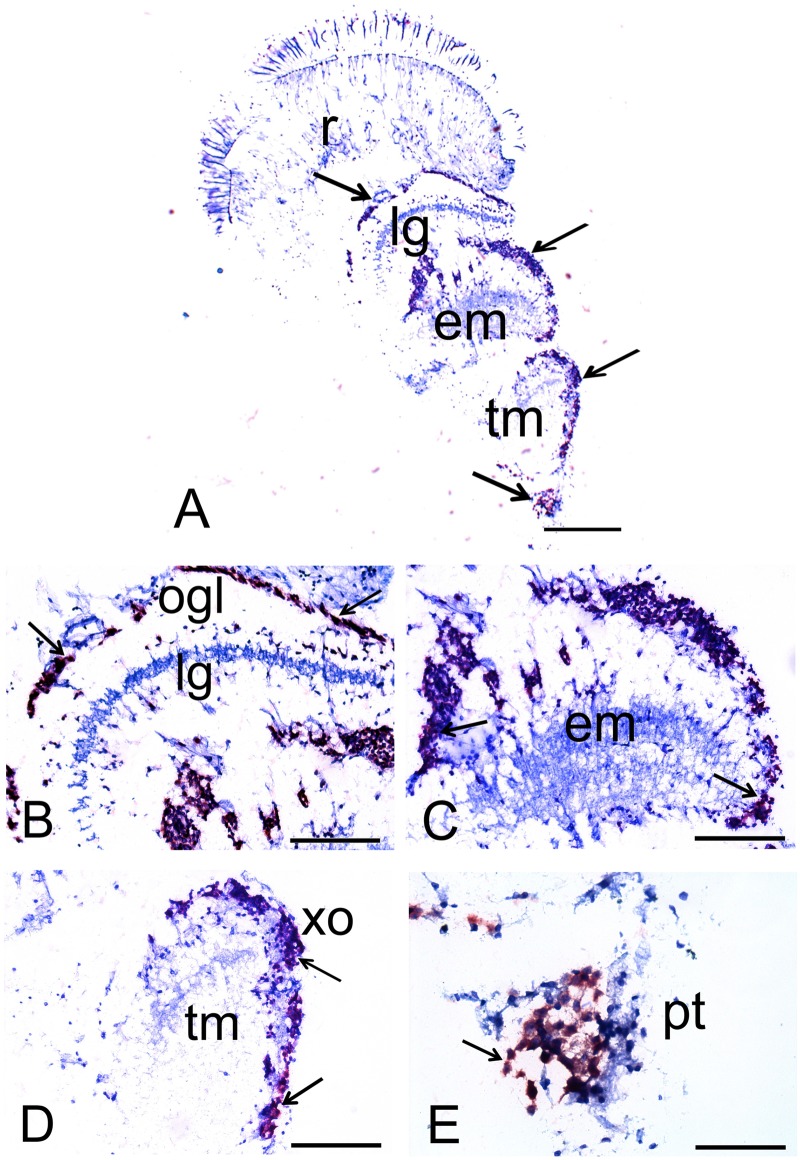
Photomicrographs showing that CHH mRNA is expressed in the eyestalk of the crayfish *P*. *clarkii* at ZT1. Panoramic view; scale bar, 400 μm (A). Amplification of lamina ganglionaris (lg); scale bar, 200 μm (B); ganglion cell layer outer (ogl). Amplification of external medulla (em); scale bar, 200 μm (C). Amplification of terminalis medulla (tm), in which you can see the XO; scale bar, 200 μm (D); and the protocerebral tract; scale bar, 100 μm (E). The arrows indicate the CHH mRNA signal in different areas in the eyestalk of crayfish.

In the optic lobe, the CHH mRNA was also localized in the lamina ganglionaris (lg), the external medulla (em), and terminalis medulla (tm) at ZT1 (Fig 2A). In the lamina ganglionaris, CHH mRNA was localized in the outer ganglion cell layer (ogl), as shown in Fig 2B. In the external (Fig 2C) medulla and terminalis, and specifically in the XO (Fig 2D) and protocerebral tract (Fig 2E), a positive CHH mRNA signal was observed in the perikarya of cells surrounding the neuropil of each of the medullas. However, no CHH signal was observed in the fibers of the neuropils or in the retinal cells.

**Fig 3:**
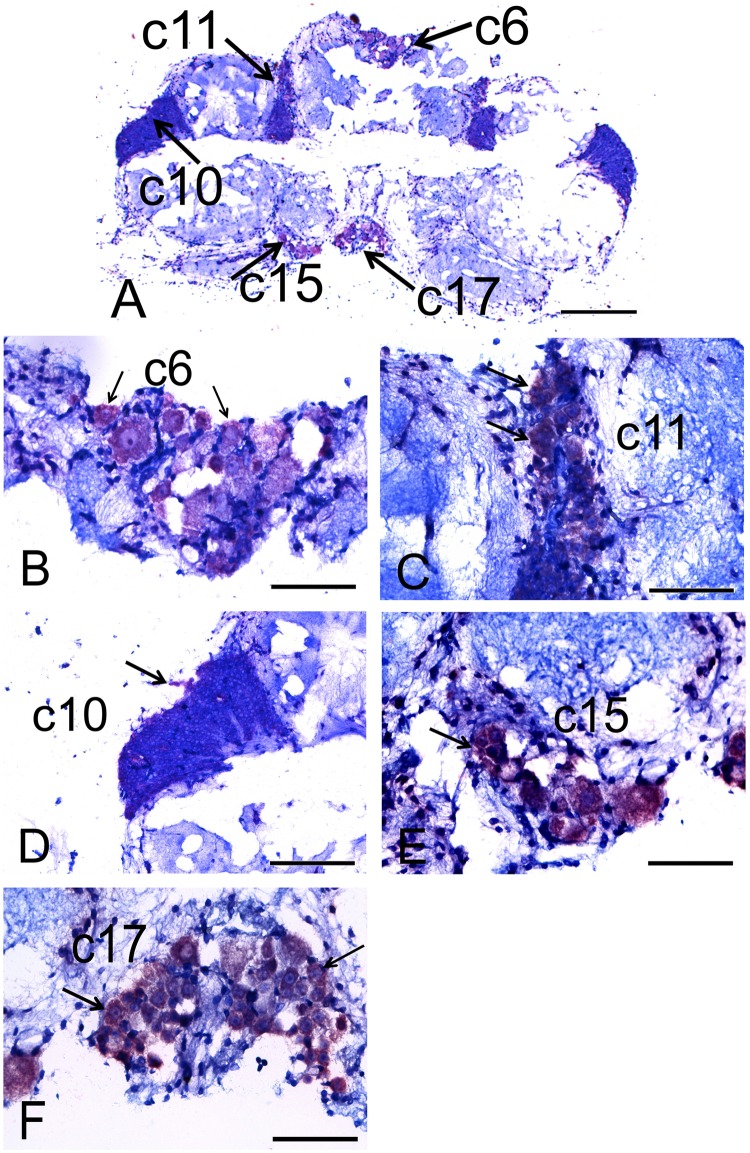
Photomicrographs showing CHH mRNA expression in the brain of the crayfish *P*. *clarkii* at ZT1. Panoramic view; scale bar, 400 μm (A). Amplification of cluster 6 (c6) of the protocerebrum (B) and cluster 11 (c11) of the deutocerebrum (C); both scale bars, 100 μm. Amplification of cluster 10 (c10) of the deutocerebrum; scale bar, 200 μm (D). Amplification of cluster 15 (c15) (E) and cluster 17 (c17) (F) of the tritocerebrum; both scale bars, 100 μm. Arrows indicate the cells in clusters that express CHH mRNA in the crayfish brain.

**Fig 4:**
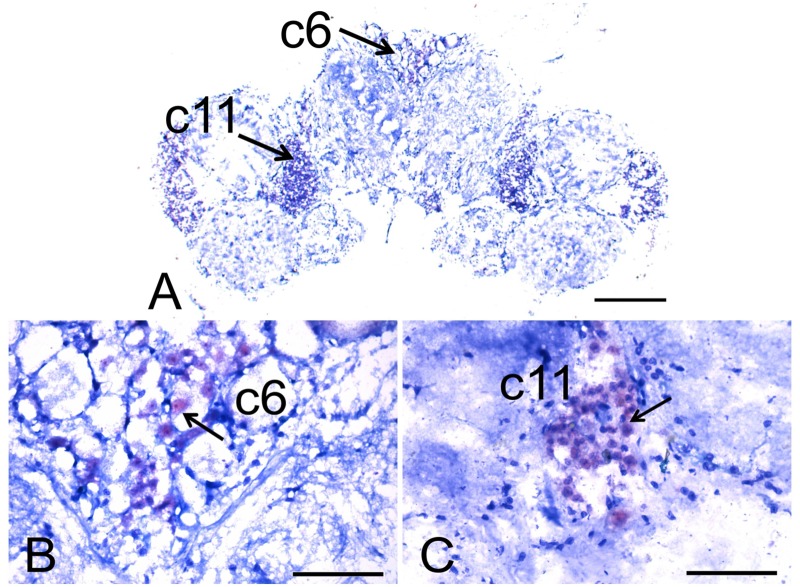
Photomicrographs showing CHH mRNA expression in the brain of the crayfish *P*. *clarkii* at ZT16. Panoramic view; scale bar, 400 μm (A). Amplification of cluster 6 (c6) of the protocerebrum (B) and cluster 11 (c11) of the deutocerebrum (C); both scale bars, 100 μm. Arrows indicate the expression of CHH mRNA in the clusters of the crayfish brain.

In the brain, CHH mRNA expression was observed in several cell clusters in the protocerebrum, deutocerebrum and tritocerebrum at both ZT1 (Fig 3A) and ZT16 (Fig 4A). At ZT1, the cell clusters (c) that were positive for CHH mRNA in the protocerebrum included c6, which contains all of the large serotonergic cells described by Sanderman et al. [24], and 14 small cells in the perikarya (Fig 3B). Those in the deutocerebrum included c11 (Fig 3C) and c10, in which some cells around the periphery were positive (Fig 3D). Those in the tritocerebrum included c15 (Fig 3E) and c17 (Fig 3F), in which CHH mRNA was always localized in the cytoplasm. At ZT16, the only cell clusters that expressed CHH mRNA were c6 of the protocerebrum, in which CHH was observed in both the cytoplasm and the nuclei of 19 small cells (Fig 4B), and in c11 of the deutocerebrum, in which CHH was observed only in the cytoplasm (Fig 4C). Despite the presence of positive signals at both ZT1 and ZT16 in c6 of the protocerebrum and c11 of the deutocerebrum, its expression level was much lower at ZT16 than at ZT1. c6 cells showed 38% positivity at ZT1 and 7% positivity at ZT16; while c11 showed 14% positivity at ZT1 and 7% positivity at ZT16 S1 Table.

Although previous studies have reported that CHH is expressed in several structures in the eyestalk, including the XO-SG complex, as well as in the tapetal cells of the retina [1], [10] and clusters 6, 14, 15 and 17 of the protocerebrum and tritocerebrum [8] of *P*. *clarkii*, it is not yet known whether CHH is synthesized in these structures because the only region in which CHH has been reported to be synthesized, stored and released is the XO-SG complex of the eyestalk [for a review, 20]. CHH mRNA levels in the brain have been shown to undergo daily circadian oscillations in both the eyestalk and brain of *P*. *clarkii* [8]. However, CHH has not been shown to be synthesized in any other structures. In this paper, the results of *in situ* hybridization show that CHH is synthesized in several structures in the eyestalk and brain of *P*. *clarkii* that showed a positive signal for CHH mRNA.

In the eyestalk, CHH mRNA was observed in the perikarya of cells surrounding the neuropil of the medullas, in the XO, as previously reported in *P*. *clarkii* [10] and other species, such as the lobster *Homarus americanus*, in which CHH has been shown to be synthesized in the XO–SG complex at both the mRNA and protein levels in both adults [25], [26] and larvae [27]. The expression of CHH has also been reported in the eyestalk of the crayfish *Orconectes limosus* [23], [28]. Based on data reported in previous studies produced by our laboratory [1], [10], [16], we expected to observe that CHH mRNA is synthesized in the retina because the crayfish retina showed anti-CHH immunoreactivity. As expected, CHH mRNA expression was observed in retinal tapetal cells (Fig 1), indicating that CHH is localized in this structure, synthesized in this region, and stored and/or released in the retina, where it contributes to a variety of metabolic functions related to eye sensitivity. No signal was observed in the optic lobe, consistent with its absence in previous reports, indicating a lack of direct neural or humoral communication between the retina and other structures, such as the optic lobe and/or brain. The finding that CHH is synthesized in tapetal cells is important because it has been reported that these cells are metabolically linked to retinal cells in addition to their functions in light reflection [29]. Our results support this hypothesis and indicate that tapetal cells could be responsible for providing metabolic support and increasing glucose levels during retinal cellular functions. Odselius and Elofsson [30] reported that in decapod crustaceans, the basement membrane of the retina forms an incomplete barrier to hemolymph. These authors suggested that the basement membrane functions to provide nutrients and regulate the transport of macromolecules and hormones into retinal cells. It has also been reported that circadian changes in CHH secretion are related to circadian changes in glucose levels in the hemolymph [31] in addition to both the eyestalk and retina [16]. It is therefore possible that CHH mRNA also shows daily or circadian changes in these structures, resulting in differences in its localization between ZT1 and ZT16.

In the brain, some cell clusters were positive for CHH, consistent with a report by Nelson-Mora et al. [8], who observed variations in CHH mRNA in the brain and immunopositivity for CHH protein in the same cell clusters in which the CHH mRNA was located, including c6 of the protocerebrum and c17 and c15 of the tritocerebrum. Recently, *in situ* hybridization in two species of crustaceans, *Eurydice pulchra* (Leach) and *Talitrus saltator* (Montagu), showed that CHH mRNA was localized in protocerebral cells [32].

The observation that CHH mRNA is expressed in structures in which it had not previously been reported is not surprising because other authors have also reported that CHH is expressed in a variety of organs in other crustacean species. A recent report characterized a novel CHH transcript and gene in the shrimp *Litopenaeus vannamei* that was identified as a type I CHH and found to be expressed in other organs in this shrimp [33]. CHH has also been shown to be present in granular cells and hemocytes [34].

Based on these results, we conclude that CHH is synthesized in several structures, including both the eyestalk and the brain. CHH may provide metabolic support and increase glucose levels to support the functions of these regions. The synthesis of CHH may vary with the circadian rhythm, resulting in daily variations in levels of the CHH protein. It is therefore necessary to conduct further studies to explore daily or circadian variations in the expression of the CHH mRNA in *P*. *clarkii*.

## Supporting information

S1 Table Percentage table. Data with which the percentages reported in the text were obtained.
(PDF)
