# Supplementary material for: Crustacean hyperglycemic hormone is synthesized in the eyestalk and brain of the crayfish Procambarus clarkii
Source: PLoS One. 2017 Apr 3;12(4):e0175046. doi: 10.1371/journal.pone.0175046 (PMC5378376; doi:10.1371/journal.pone.0175046)
Supplement: S1 Table — Data with which the percentages reported in the text were obtained. (PDF) [file pone.0175046.s001.pdf]

Total tissue area			
Figure 3A	Figure 3B	Figure 4A	Figure 4B
2598395	4628274	4628070	4633560
149858	11349038	995439	897688
**2448537**	**3980535**	**3632631**	**3735872**

Area CHH mRNA expression			
Figure 3A	Figure 3B	Figure 4A	Figure 4B
76708	484590	111451	35001
76708	88624	65847	61008
76708		69009	146579
76708		20850	17746
76708		4299	8472
76708			
76708			
76708			
76708			
76708			
76708			
76708			
**920496**	**573214**	**271456**	**268806**

Figure	Total tissue area	Area CHH mRNA expression	% positivity CHH
3A	**2448537**	**920496**	**37.59**
3B	**3980535**	**573214**	**14.4**
4A	**3632631**	**271456**	**7.47**
4B	**3735872**	**268806**	**7.19**
